# The glycine-containing dipeptide leucine-glycine raises accumbal dopamine levels in a subpopulation of rats presenting a lower endogenous dopamine tone

**DOI:** 10.1007/s00702-022-02487-4

**Published:** 2022-03-24

**Authors:** Yasmin Olsson, Helga Lidö, Mia Ericson, Bo Söderpalm

**Affiliations:** 1 Addiction Biology Unit, Department of Psychiatry and Neurochemistry, Institute of Neuroscience and Physiology, Sahlgrenska Academy, University of Gothenburg, PO Box 410, 405 30 Gothenburg, Sweden; 2 Beroendekliniken, Sahlgrenska University Hospital, Gothenburg, Sweden; 3 Department of Neurology, Sahlgrenska University Hospital, Gothenburg, Sweden

**Keywords:** Dopamine, Glycine receptor, Alcohol, Nucleus accumbens, In vivo microdialysis, Locomotion

## Abstract

Interventions that elevate glycine levels and target the glycine receptor (GlyR) in the nucleus Accumbens (nAc) reduce ethanol intake in rats, supposedly by acting on the brain reward system via increased basal and attenuated ethanol-induced nAc dopamine release. Glycine transport across the blood brain barrier (BBB) appears inefficient, but glycine-containing dipeptides elevate whole brain tissue dopamine levels in mice. This study explores whether treatment with the glycine-containing dipeptides leucine-glycine (Leu-Gly) and glycine-leucine (Gly-Leu) by means of a hypothesized, facilitated BBB passage, alter nAc glycine and dopamine levels and locomotor activity in two rodent models. The acute effects of Leu-Gly and Gly-Leu (1–1000 mg/kg, i.p.) alone or Leu-Gly in combination with ethanol on locomotion in male NMRI mice were examined in locomotor activity boxes. Striatal and brainstem slices were obtained for ex vivo HPLC analyses of tissue levels of glycine and dopamine. Furthermore, the effects of Leu-Gly i.p. (1–1000 mg/kg) on glycine and dopamine output in the nAc were examined using in vivo microdialysis coupled to HPLC in freely moving male Wistar rats. Leu-Gly and Gly-Leu did not significantly alter locomotion, ethanol-induced hyperlocomotor activity or tissue levels of glycine or dopamine, apart from Gly-Leu 10 mg/kg that slightly raised nAc dopamine. Microdialysis revealed no significant alterations in nAc glycine or dopamine levels when regarding all rats as a homogenous group. In a subgroup of rats defined as dopamine responders, a significant elevation of nAc dopamine (20%) was seen following Leu-Gly 10–1000 mg/kg i.p, and this group of animals presented lower baseline dopamine levels compared to dopamine non-responders. To conclude, peripheral injection of glycine-containing dipeptides appears inefficient in elevating central glycine levels but raises accumbal dopamine levels in a subgroup of rats with a lower endogenous dopamine tone. The tentative relationship between dopamine baseline and ensuing response to glycinergic treatment and presumptive direct interactions between glycine-containing dipeptides and the GlyR bear insights for refinement of the glycinergic treatment concept for alcohol use disorder (AUD).

## Introduction

A central mechanism for how alcohol and other drugs of abuse elicit reward revolves around their actions on the mesolimbic dopamine system (Di Chiara and Imperato 1988; Koob and Volkow 2016). For individuals susceptible to Alcohol Use Disorder (AUD), adaptations in this neurocircuitry appear to entail a transition from controlled, occasional alcohol intake into a compulsion to drink (Koob and Volkow 2016). In both rodent and human models, enhanced dopamine activity in the nucleus accumbens (nAc) in the ventral striatum, a terminal area of the mesolimbic dopamine system, has been linked to the positive reinforcing properties of alcohol (Di Chiara and Imperato 1988; Boileau et al. 2003; Gonzales et al. 2004; Yoder et al. 2007). Conversely, attenuated accumbal dopamine output characterizes the negative emotional state associated with alcohol withdrawal (Rossetti et al. 1992; Volkow et al. 2007; Koob and Volkow 2016). Advances in the neuroscience of drug reward and addiction have endowed us with an in-depth understanding for how several drugs that are abused by humans activate the brain reward system (Nestler 2005, Volkow et al. 2019). This line of research is pivotal to the field of addiction medicine since it may yield new pharmacological targets for the treatment of AUD and other substance use disorders. Attempts to precisely delineate the neurobiological underpinnings of alcohol reward are currently ongoing and evidence from animal studies suggests that alcohol enters the mesolimbic reward pathway by targeting GlyRs in the nAc, among other receptor populations (Molander and Söderpalm 2005b, Spanagel 2009; Soderpalm et al. 2017). Accordingly, both local (into the nAc) and systemic treatment with the endogenous GlyR agonist glycine or glycine-transporter-1(GlyT-1)-inhibitors, that regulate CNS glycine levels, elevate basal dopamine levels in a sub-population of rats denoted dopamine responders, attenuate the alcohol-induced dopamine elevation and robustly reduce alcohol intake in the rat (Molander and Söderpalm, 2005b, Molander et al. 2007; Lidö et al. 2009, 2012; Vengeliene et al. 2010, 2018; Olsson et al. 2020). Thus, compounds that elevate central glycine levels and target the GlyR appear to interfere with the reinforcing properties of alcohol and may represent a new pharmacologic treatment principle for AUD (Soderpalm et al. 2017; Olsson et al. 2020).

Nevertheless, treatment protocols for glycine, utilizing either glycine’s agonistic effect on the GlyR in AUD animal models or its co-agonistic effect on the NMDA-receptor in human studies for the treatment of schizophrenia, require impractically large doses of glycine (Heresco-Levy et al. 1999; Buchanan et al. 2007; Olsson et al. 2020). In addition, serum and brain glycine levels as measured by magnetic resonance spectroscopy (MR-S) vary considerably between subjects after intravenous glycine treatment (Kaufman et al. 2009). This may be related to impeded blood brain barrier (BBB) passage for glycine, a free amino acid, compared to e.g. dipeptides (Toth and Lajtha 1981; Hawkins et al. 2006; Tanaka et al. 2019). Leucine-Glycine (Leu-Gly) and Glycine-Leucine (Gly-Leu) are two glycine-containing dipeptide isomers that in a recently published study have been demonstrated to elevate whole brain dopamine levels ex vivo following systemic administration in a mouse model (Han et al. 2018). Moreover, Leu-Gly and Gly-Leu elicited enhanced endurance in mice running on a treadmill, possibly a result of increased locomotion, that is in turn a well-known proxy for substances that elevate central dopamine levels (Carlsson et al. 1974; Liljequist et al. 1981; Wise and Bozarth 1987; Han et al. 2018). We hypothesize that the effects of Leu-Gly and Gly-Leu stem from their glycine constituents and involve GlyRs in the nAc. Cerebral endothelial cells constituting the BBB participate actively in regulating the composition of brain interstitial fluid (Abbott et al. 2006; Hawkins et al. 2006). Transporter proteins scattered throughout the surfaces of the BBB enable passage of amino acids (Abbott et al. 2006; Hawkins et al. 2006). Leucine, an essential, neutral amino acid is mainly transported over the BBB by means of the L-type amino acid transporter 1 (LAT1)-system whereas the non-essential, neutral amino acid glycine is employing the less efficient system y + (Oxender and Christensen 1963; Hawkins et al. 2006). It is conceivable that BBB passage may be facilitated if glycine is anchored onto a molecule that passes through the LAT1-system, e.g. leucine. The LAT1-system has a broad substrate specificity, in addition to naturally occurring amino acids, it also transports dipeptides and amino acid-related compounds such as L-DOPA and gabapentin (Uchino et al. 2002; Tanaka et al. 2019).

The aim of this study was to, for the first time to our knowledge, examine whether systemic treatment with glycine-containing dipeptides (1) alter locomotion and ethanol-induced locomotor activity in mice, (2) alter ex vivo accumbal glycine and dopamine content in mice and (3) elevate in vivo extracellular levels of glycine and dopamine in rat nAc. We hypothesized that systemic treatment with Leu-Gly would elevate striatal glycine and dopamine levels due to its glycine content and presumable GlyR-mediated dopamine response, in line with its documented effects on whole brain dopamine levels (Han et al. 2018). Moreover, we hypothesized that systemic treatment with Leu-Gly would attenuate the ethanol-induced locomotor activity, as an indirect measure of mitigated central dopamine elevation in response to ethanol.

## Materials and methods

### Animals

For locomotor activity and ex vivo neurochemistry studies, a total number of 152 male NMRI mice weighing 18–25 g at arrival were supplied by Janvier (France). For in vivo microdialysis, a total number of 54 male Wistar rats weighing 260–280 g at arrival were supplied by Envigo (Netherlands). Mice were housed eight to a cage whereas rats were housed four to a cage prior to surgery and single-housed after surgery, under controlled environmental conditions comprising constant room temperature of 20–22 °C, humidity of 50–65% and regular light-dark conditions with lights on at 07:00 a.m. and off at 07:00 p.m. Before any experimental procedures were initiated, animals were allowed at least one week of acclimatization to the facilities. All animals had access to regular chow (Harlan Teklad, England) and tap water ad libitum. The experiments were approved by the Ethics Committee for Animal Experiments, Gothenburg, Sweden.

### Drugs

Leu-Gly and Gly-Leu (Bachem, Switzerland) were dissolved in 0.9% NaCl solution to a desired dose of 1, 10, 100 or 1000 mg/kg and administered i.p. in a volume of 2.0 ml/kg. In previous studies of Leu-Gly isomers in mice, ex vivo dopamine levels in whole brain were elevated after treatment with either 1 or 10 mg/kg i.p. of either isomer (Han et al. 2018). In this explorative study, we chose also to include a 10- and 100-fold higher dose in which the latter dose approximately corresponds to a molar content of glycine that upon systemic administration produces a trend for raised accumbal glycine levels between 50 and 150% (Olsson et al. 2020). Ethanol (95%; Kemetyl AB, Sweden) was diluted in 0.9% NaCl to a concentration of 15% for i.p. injections corresponding to a dose of 2.5 g/kg. This dose of ethanol has repeatedly been shown to induce increased locomotor activity in mice, e.g. (Carlsson et al. 1974; Ulenius et al. 2019).

### Locomotor activity

**Fig. 1:**
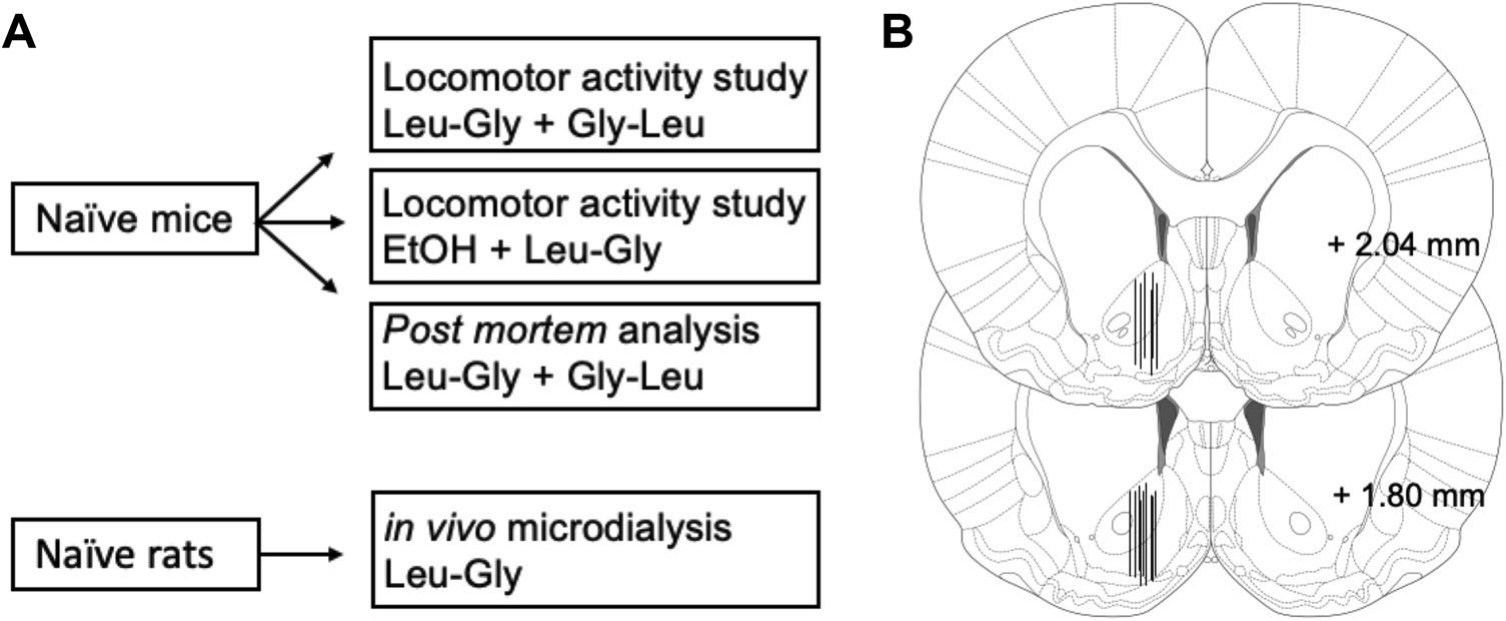
Overview and probe placement verification. **A** Schematic overview of the experimental design. **B** Location of 18 representative microdialysis probes in the nAc, as depicted by black lines. The active space of the probe (2 mm of its inferior aspect) targets the nAc core–shell border region. Numbers on the right side of the figure represent distance from bregma

The locomotor activity studies were performed as previously described (Ulenius et al. 2019). In brief, the mice were monitored for a total time period of 2 hours including 1 hour of habituation to the monitoring box prior to acute i.p injections with Leu-Gly or Gly-Leu, vehicle and/or ethanol. The underlying rationale being that the ethanol-induced mesolimbic dopamine elevation is, in part, mediated by accumbal GlyRs and that glycine administered exogenously might block this effect. Locomotor activity was measured using identical sound attenuated, ventilated and dim lit locomotor activity boxes equipped with a two-layer grid, consisting of rows of photobeams (40 × 40 cm, Med Assoc., USA), which allowed a computer-based monitoring system (Activity Monitor 7, Med Assoc., USA) to register ambulatory counts. Ambulatory counts were defined as the total number of consecutive photobeams interrupted during a time period of 5 minutes and were measured during the entire experiment. The choice of mice, rather than rats, for locomotor activity studies was based on (1) elevated whole brain dopamine levels in mice after treatment with Leu-Gly isomers, (2) repeated and robust findings demonstrating increased locomotion in response to ethanol in mice, that is associated with its effects on the mesolimbic dopamine system and (3) the notion that alcohol produces a biphasic effect of both stimulatory (increased dopaminergic and noradrenergic activity) and sedative (increased GABAergic activity) behavior where the former may be occluded by the latter effects on locomotion in rats and more reliably separated in mice (Carlsson et al. 1974; Frye and Breese 1981; Wise and Bozarth 1987; Engel et al. 1988; Han et al. 2018). A schematic overview of all experiments is provided in Fig. 1A.

### Post mortem analysis

In analogy with a previous study on the effects of Leu-Gly and Gly-Leu on whole brain dopamine levels ex vivo, mice were chosen for the present post-mortem analyses (Han et al. 2018). The mice were sacrificed 30 min after receiving randomly assigned i.p. injections with Leu-Gly, Gly-Leu or vehicle, a timepoint approximately corresponding to when a trend for elevated glycine and dopamine levels were present in the microdialysis experiments in rats. Following decapitation, the brains were removed and GlyR-containing areas of interest, i.e. the nAc, the dorsal striatum and the brainstem, were rapidly punched-out on a cold glass plate, immediately frozen on dry ice and kept frozen at − 80 °C until subsequent biochemical analyses. Next, the brain tissue samples were homogenized by ultrasound homogenization in a solution of 0.1 M perchloric acid and 10% EDTA. The homogenate was centrifuged at 12 000 rpm for 10 min at 4 °C and the supernatant was collected and analyzed for glycine and dopamine using a high-performance liquid chromatography (HPLC) with an electrochemical cell detector as previously described (Ulenius et al. 2020).

### In vivo microdialysis

The rats were anesthetized with 4% isoflurane (Baxter, Sweden), mounted onto a stereotactic instrument (David Kopf Instruments Tujunga, CA, USA) and placed on a heating pad to prevent hypothermia during surgery. Three holes were drilled for the placement of a probe and two anchoring screws. An I-shaped, custom-made probe with a semi-permeable membrane was gently lowered into the nAc core–shell borderline region (A/P: + 1.85, M/L: − 1.4, D/V: − 7.8 mm relative to bregma and dura, coordinates from Paxinos and Watson 2007. The core–shell border region was targeted since this subregion of the nAc is associated with dopamine elevation upon ethanol intake (Howard et al. 2009). The 2 mm active space of the probe refers to the exposed length of the membrane, where the fluid exchange between perfused and extracellular fluid takes place. Probes and anchoring screws were fixed to the skull using Harvard cement (DAB Dental AB, Stockholm, Sweden). Marcain^®^ (bupivacaine, AstraZeneca, Sweden) was applied locally for analgesic purposes and 2 ml NaCl 0.9% was injected subcutaneously to prevent dehydration. After surgery, the rats were single housed and allowed to recover for two days prior to initiating the microdialysis experiments.

On the experimental day, the probes were connected to a microperfusion pump (U-864 Syringe Pump, AgnTho’s, Sweden) via a swivel allowing the animal to move around freely. The probes were perfused with Ringer solution for 2 h prior to sampling to obtain a balanced fluid exchange before baseline sampling began. During the entire experiment, the infusion rate for the Ringer solution was set at 2 μl/min and dialysate samples (40 μl) were collected every 20 min. When at least three consecutive stable values of dopamine were obtained, systemic treatment with Leu-Gly or vehicle was initiated. Baseline levels for each animal was set to 100%. Microdialysate dopamine content was separated and quantified using HPLC with an electrochemical cell detector as previously described (Ulenius et al. 2020). An external standard containing 3.25 nM of dopamine was used to identify the dopamine peak and to quantify dopamine concentrations in the dialysates. All samples of dopamine were analyzed online and the remainder of the dialysate was preserved in sodium azide and stored at a cold temperature for later analysis of glycine in a different HPLC set up with fluorescence detection. External standards containing 500 nM and 1000 nM of glycine were used for glycine analysis. Rats were sacrificed directly after the experiment, brains were removed, and probe placements were verified using a vibroslicer (Campden Instruments Ltd., USA). Rats with incorrect probe placement and/or substantial hemorrhage around the probe were excluded. Probe placement is illustrated in Fig. 1B. Rats rather than mice were used to more accurately compare the present findings to results obtained in previous microdialysis studies on glycinergic compounds that also employed rats. Moreover, microdialysis in mice confers technical challenges due to its smaller size. For example, the probe insertion may cause damage to a relatively larger part of the mouse brain, making data obtained in rat microdialysis studies more reliable and the preferred choice for most research groups performing brain microdialysis studies.

### Statistics

Locomotor activity measurements over time (*t* = 0–60 min) were evaluated by a two-way analysis of variance (ANOVA) followed by Tukey’s multiple comparison test when applicable. Glycine and dopamine data derived from mice post-mortem analysis were evaluated by a one-way ANOVA followed by Dunnett’s post hoc. Extracellular glycine and dopamine measurements over time (*t* = 0–120 min) from in vivo microdialysis were evaluated by a two-way ANOVA followed by Dunnett’s multiple comparison test whereas area under the curve (AUC) data were analyzed using one-way ANOVA. In the microdialysis study, rats were classified as dopamine responders if dopamine was elevated more than 10% compared to baseline within the first hour after injection, in line with previous subdivision for rats that received local (into the nAc) or systemic treatment with glycine (Molander and Soderpalm, 2005a, Olsson et al. 2020). Baseline glycine and dopamine levels and peak glycine levels for dopamine responders vs non-responders were compared using an independent samples *t* test. Relationships between peak glycine and AUC dopamine were examined with Pearson’s correlation test. All values are presented as mean ± SEM. A probability value (*p*) < 0.05 was considered statistically significant. Statistics were performed using GraphPad Prism, version 9.0.1 for Mac OS (GraphPad Software Inc., USA).

## Results

### Glycine-containing dipeptides do not alter basal locomotor activity nor attenuate the ethanol-induced hyperlocomotion in mice

**Fig. 2:**
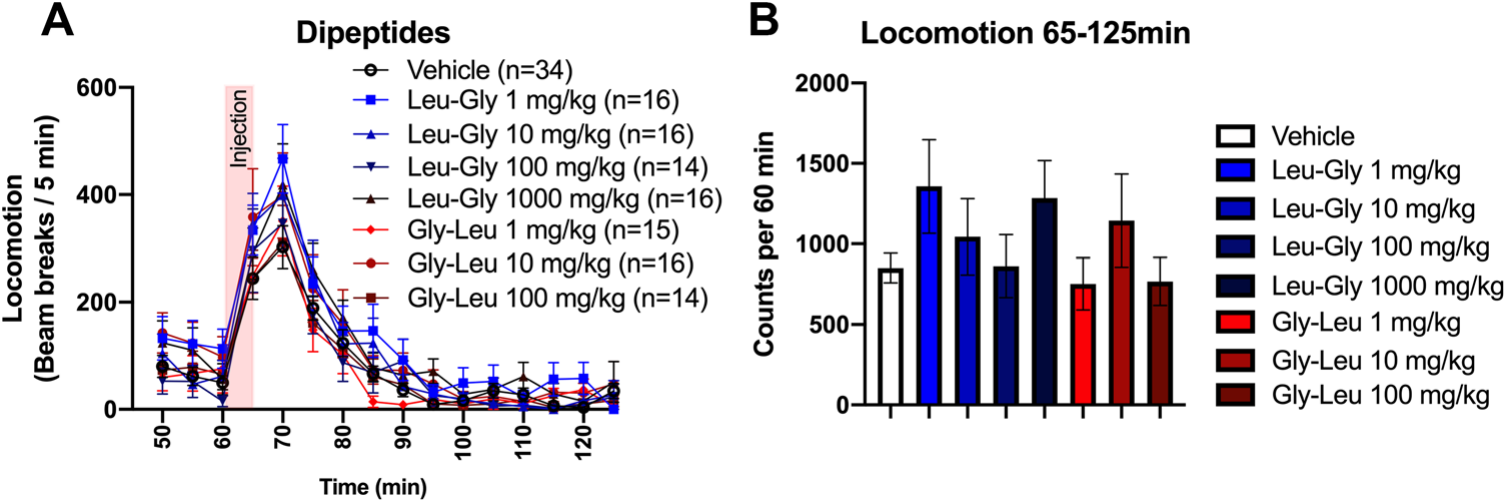
Effects of Leu-Gly and Gly-Leu on locomotor activity. (**A**–**H**) Systemic administration of Leu-Gly in four different doses and Gly-Leu in three different doses does not alter locomotor activity compared to vehicle-treated controls. Data are presented as mean values ± SEM, *n* number of mice

**Fig. 3:**
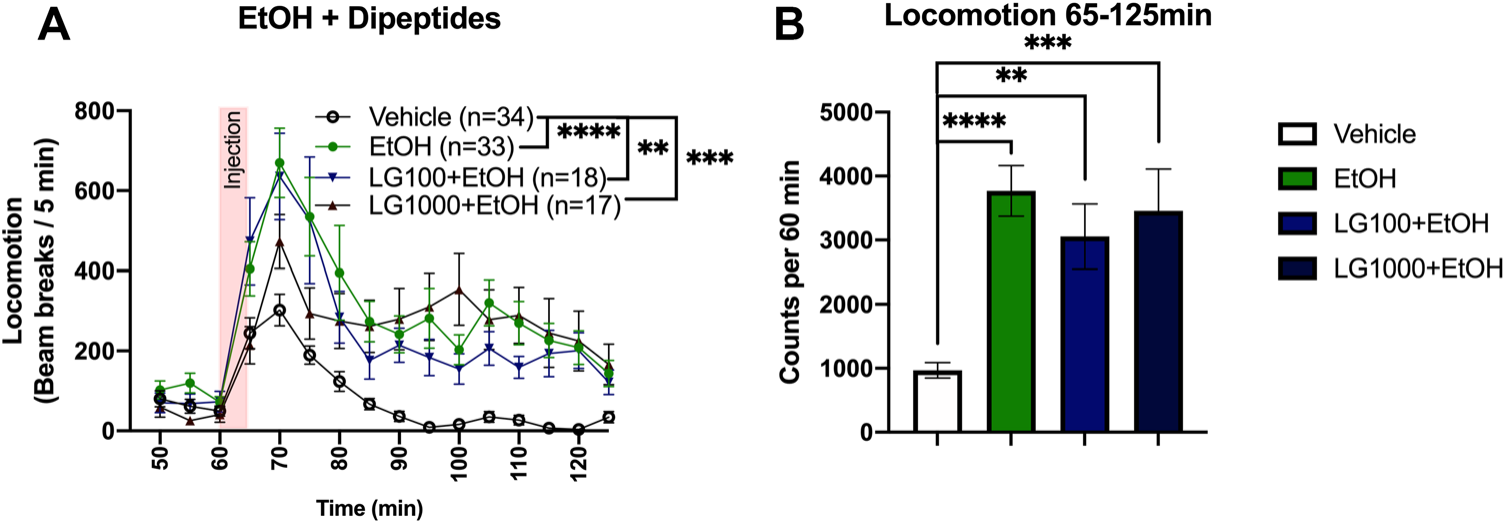
Effects of Leu-Gly on EtOH-induced hyperlocomotor activity. (**A**–**H**) Acute administration of EtOH significantly increased the locomotor activity as compared to vehicle-treated controls. Combined treatment with Leu-Gly (100 or 1000 mg/kg) did not attenuate the EtOH-induced hyperlocomotion. Data are presented as mean values ± SEM, **p < .01, ***p < .001, ****p < .0001, *n* number of mice

Mice receiving acute, systemic treatment with four different doses of Leu-Gly or three different doses of Gly-Leu did not present significantly altered locomotor activity as compared to vehicle-treated controls (Fig. 2). Since locomotor activity did not differ between the two dipeptide isomers, the remaining studies, with exception for the post-mortem analysis, were performed using only Leu-Gly. To explore tentative interactive effects of glycine-containing dipeptides and ethanol in the locomotor activity model, mice in a next step received i.p. injections with both ethanol and/or one of the two highest doses of Leu-Gly, i.e. 100 or 1000 mg/kg. As expected and depicted in Fig. 3A, ethanol alone significantly increased locomotor activity, two-way ANOVA_*t*=65–125_; treatment effect *F*_(398)_ = 14.52, *p* < 0.0001, time effect *F*_(11,1078)_ = 19.36, *p* < 0.0001, interaction term *F*_(33,1078)_ = 1.32, *p* = 0.111; Tukey’s post hoc vehicle vs EtOH; *p* < 0.0001. However, in contrary to what was hypothesized, the addition of Leu-Gly 100 or 1000 mg/kg did not attenuate the ethanol-induced hyperlocomotion; vehicle *vs* EtOH + LG100; *p* = 0.001, vehicle vs EtOH + LG1000; *p* = 0.0001. These effects were also evident upon comparison of AUCs, one-way ANOVA; *F*_(3100)_ = 13.74, *p* < 0.0001, Tukey’s post hoc vehicle vs EtOH; *p* < 0.0001; vehicle *vs* EtOH + LG100; *p* = 0.0019; vehicle *vs* EtOH + LG1000; *p* = 0.0002 (Fig. 3B).

### Glycine-containing dipeptides produce minor effects on tissue levels of accumbal dopamine but do not alter brain tissue levels of glycine in mice

**Fig. 4:**
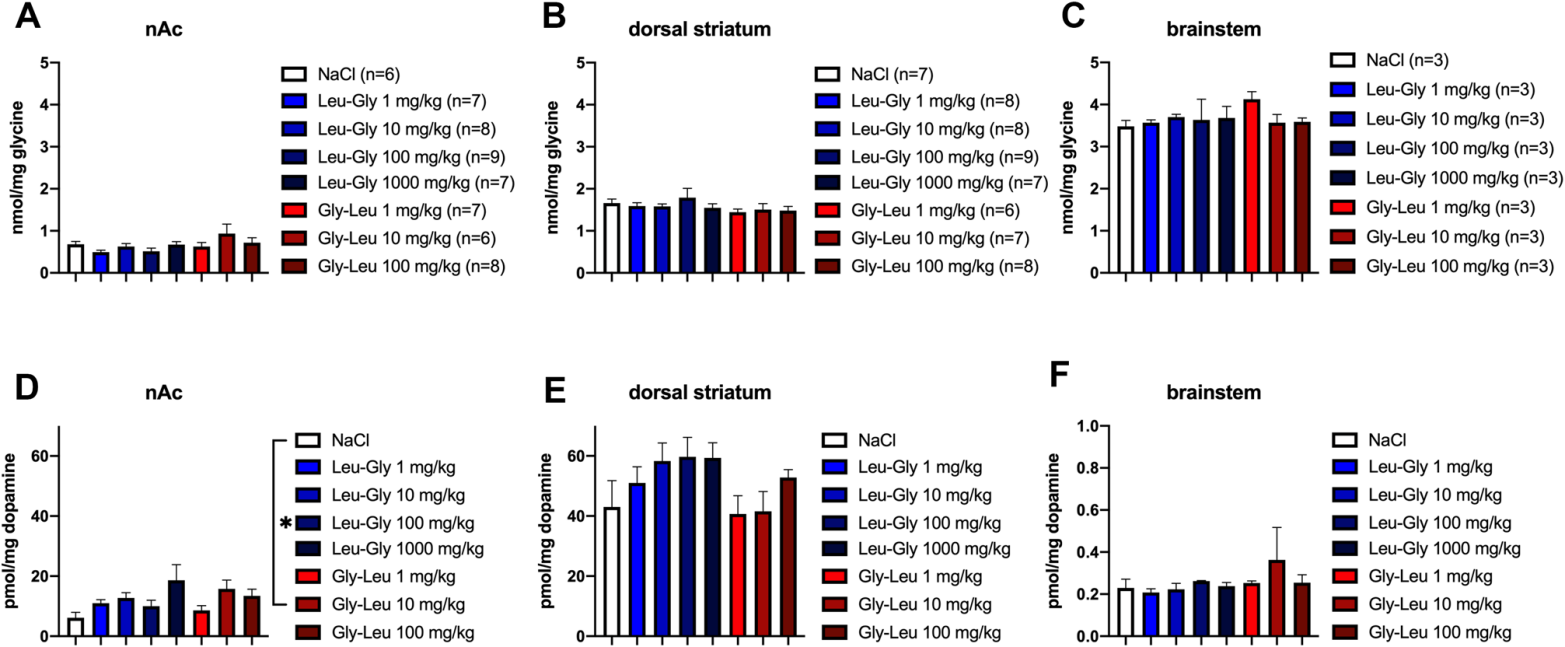
Effects of Leu-Gly and Gly-Leu on glycine and dopamine tissue levels ex vivo in nAc, dorsal striatum and brainstem. (**A**–**C**) Systemic administration of Leu-Gly in four different doses and Gly-Leu in three different doses did not alter glycine tissue levels in the nAc, dorsal striatum (ds) or brainstem as compared to vehicle-treated controls. **D** Following treatment with the intermediate dose of Gly-Leu, accumbal dopamine tissue levels were significantly elevated, whereas the remaining doses of Gly-Leu or any dose of Leu-Gly did not significantly alter dopamine levels. (**E**–**F**) Dopamine levels in the dorsal striatum and brainstem were not significantly altered by treatment with Leu-Gly or Gly-Leu. Data are presented as mean values ± SEM, *n* number of mice

A post-mortem analysis was performed to confirm previous results from whole brain tissue analysis but this time refining the analyses to dopamine end-terminal regions or regions with a high density of GlyRs (Lynch 2004; Björklund and Dunnett 2007; Soderpalm et al. 2017). Mice receiving acute systemic treatment with four different doses of Leu-Gly or three different doses of Gly-Leu were sacrificed and glycine and dopamine tissue levels ex vivo were analyzed from nAc, dorsal striatum and brainstem tissue. As depicted in Fig. 4A–C, glycine tissue levels ex vivo were not significantly altered in response to treatment with Leu-Gly or Gly-Leu, as compared to vehicle-treated controls. In contrast, treatment with Gly-Leu, but not Leu-Gly produced a significant increase in tissue levels of dopamine in the nAc, one-way-ANOVA; *F*_(3,24)_ = 3.83, *p* = 0.023. A subsequent Dunnett’s post hoc test revealed a significant effect for the intermediate dose of Gly-Leu, vehicle *vs* Gly-Leu 10 mg/kg; *p* = 0.017 (Fig. 4D). No significant alterations in dopamine levels were observed in neither the dorsal striatum nor in the brainstem (Fig. 4E–F).

### Leu-Gly did not significantly alter neither extracellular glycine nor dopamine levels in the rat nAc when regarding all animals as a homogenous group

**Fig. 5:**
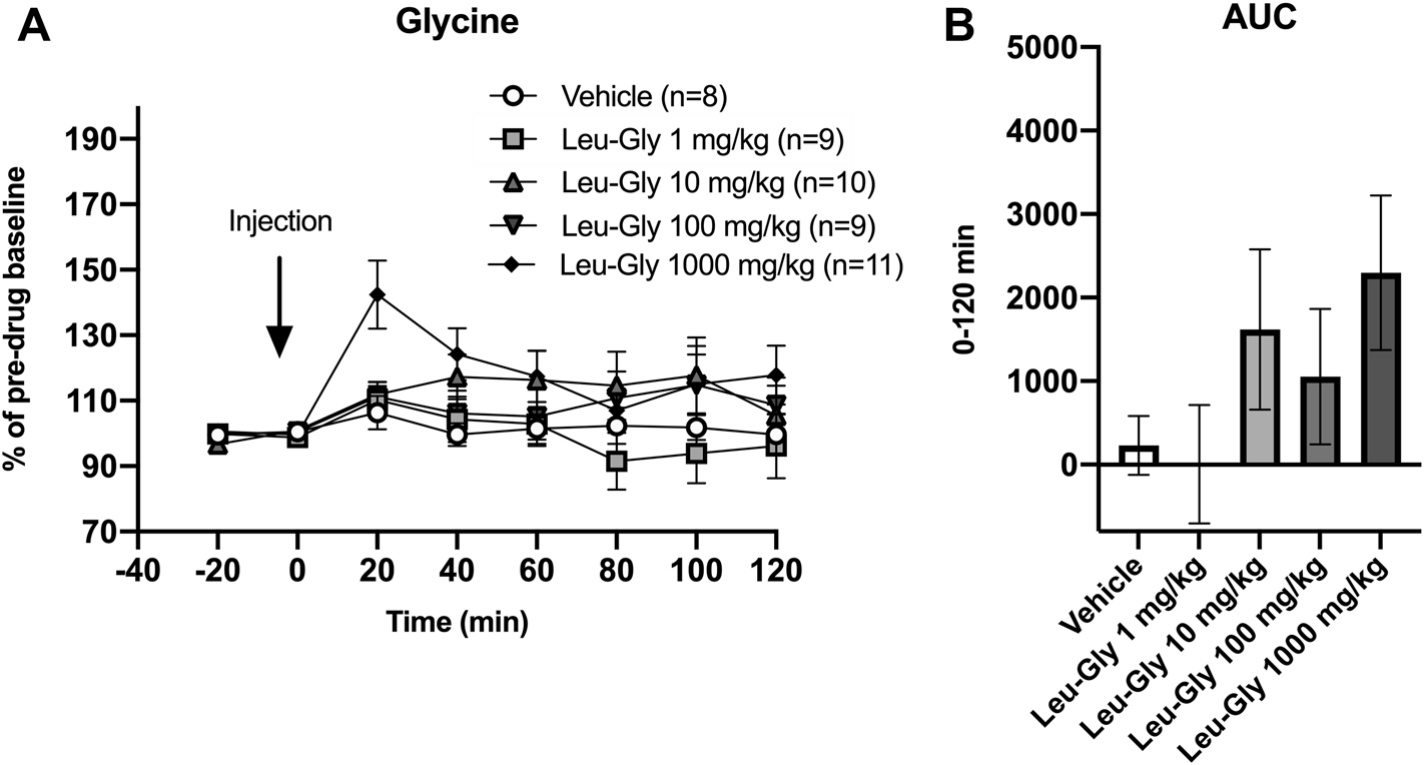
Effects of Leu-Gly on accumbal glycine levels in vivo. **A** Following systemic administration of Leu-Gly i.p. in four different doses, a trend toward elevated accumbal glycine levels was observed at timepoint 20’ for the highest dose. **B** Comparisons of AUCs failed to reveal any significant alterations of accumbal glycine levels. Shown are mean values ± SEM, *n* number of rats

**Fig. 6:**
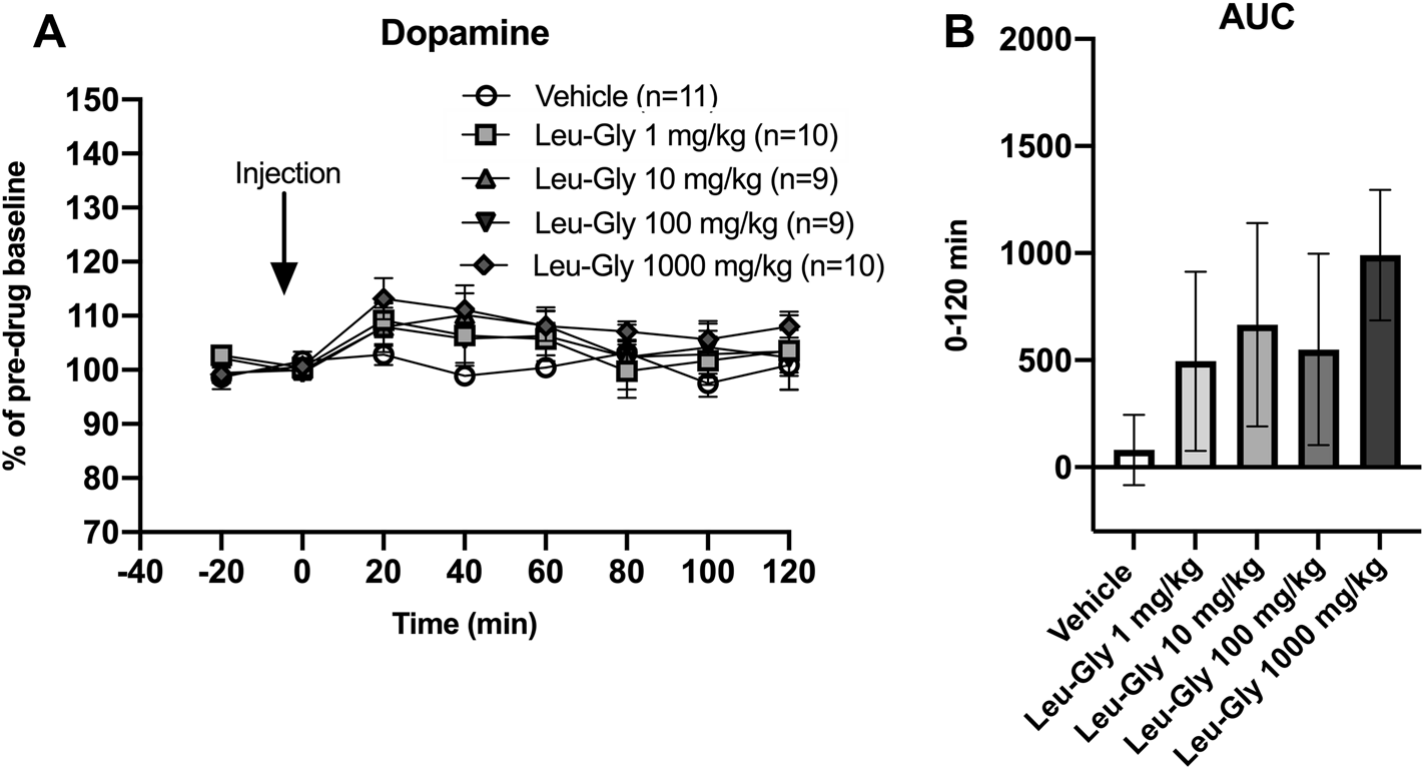
Effects of Leu-Gly on accumbal dopamine levels in vivo. **A** Systemic administration of Leu-Gly in four different doses did not significantly alter accumbal dopamine levels, as compared to vehicle-treated controls. **B** Comparisons of AUC’s did not reveal a significant elevation of accumbal dopamine levels. Shown are mean values ± SEM, *n* number of rats

Systemic treatment with Leu-Gly in four different doses did not significantly alter accumbal glycine output, two-way ANOVA_*t*=0–120_; treatment effect *F*_(431)_ = 2.04, *p* = 0.114, time effect *F*_(6186)_ = 4.20, *p* < 0.001, interaction term *F*_(24,186)_ = 1.66, *p* = 0.033, (Fig. 5A). Following the highest dose of Leu-Gly, a non-significant trend toward elevated accumbal glycine levels (approx. 40%) was observed at timepoint 20 min post injection, Dunnett’s multiple comparisons test; Leu-Gly 1000 mg/kg vs vehicle *p* = 0.324. Comparisons of AUCs did not reveal any significant alterations of accumbal glycine levels (Fig. 5B). Likewise, none of the treatment groups demonstrated any significant alterations on accumbal dopamine levels (Fig. 6A-B).

### Extracellular dopamine levels were significantly raised in a subgroup of rats that presented a lower endogenous dopamine tone

**Fig. 7:**
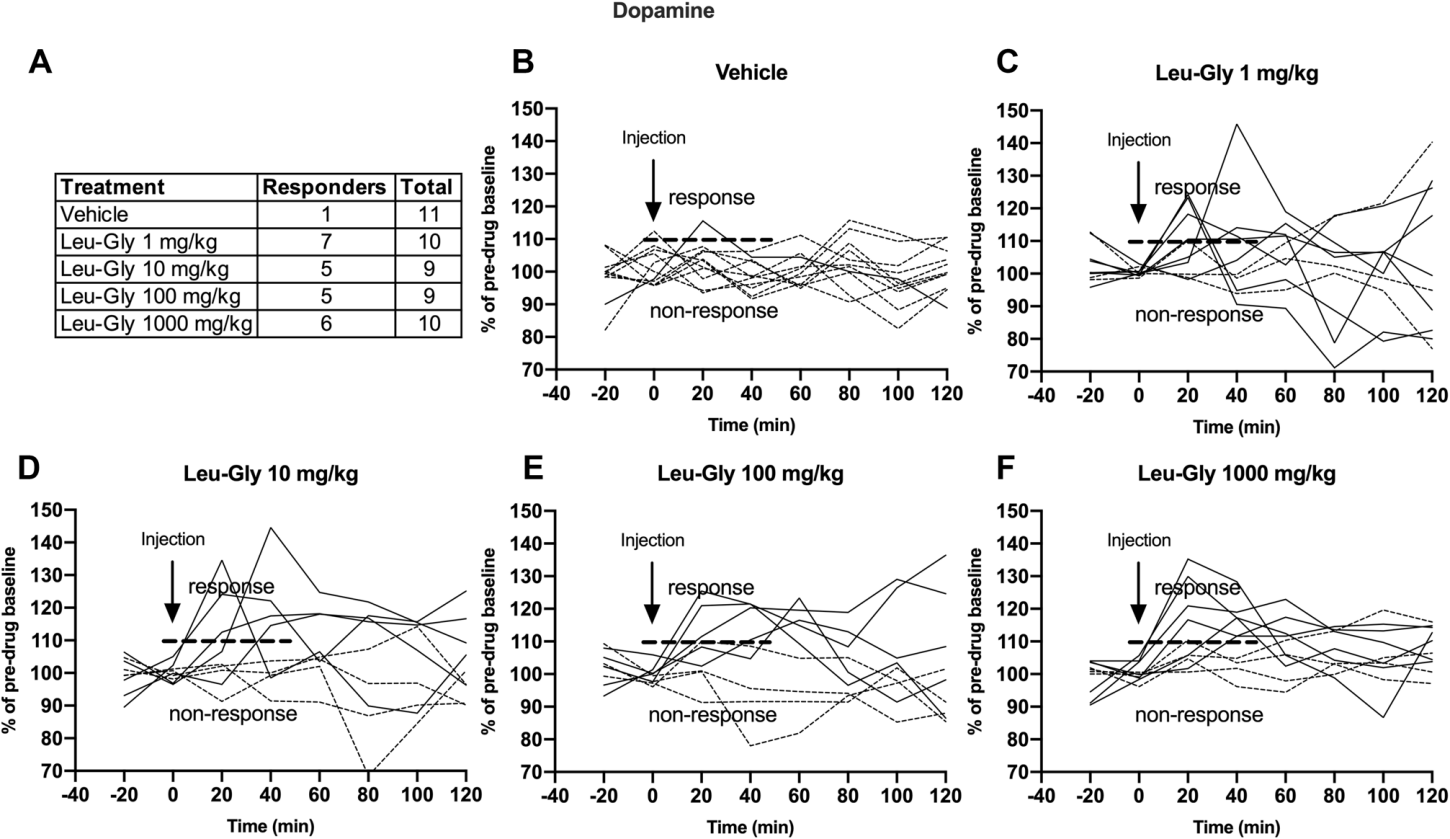
Distribution of dopamine responding rats. **A** Distribution of individual rats responding with an accumbal dopamine elevation after systemic treatment with Leu-Gly, divided by treatment group. A dopamine response was defined as an elevation of accumbal dopamine levels by more than 10% from baseline during the first hour after injection. (**B**–**F**) Each curve represents the accumbal dopamine response for an individual rat after treatment with vehicle, Leu-Gly 1 mg/kg, 10 mg/kg, 100 mg/kg or 1000 mg/kg i.p. The solid line represents rats classified as responders whereas the dotted line represents animals classified as non-responders. Rats presenting a dopamine elevation above 10% compared to baseline during the first hour after injection were classified as dopamine responders. A similar proportion of responders were identified among the four different active treatment groups; Leu-Gly 1 mg/kg: 7 out of 10 rats, 10 mg/kg: 5 out of 9 rats, 100 mg/kg: 5 out of 9 rats and 1000 mg/kg: 6 out of 10 rats

**Fig. 8:**
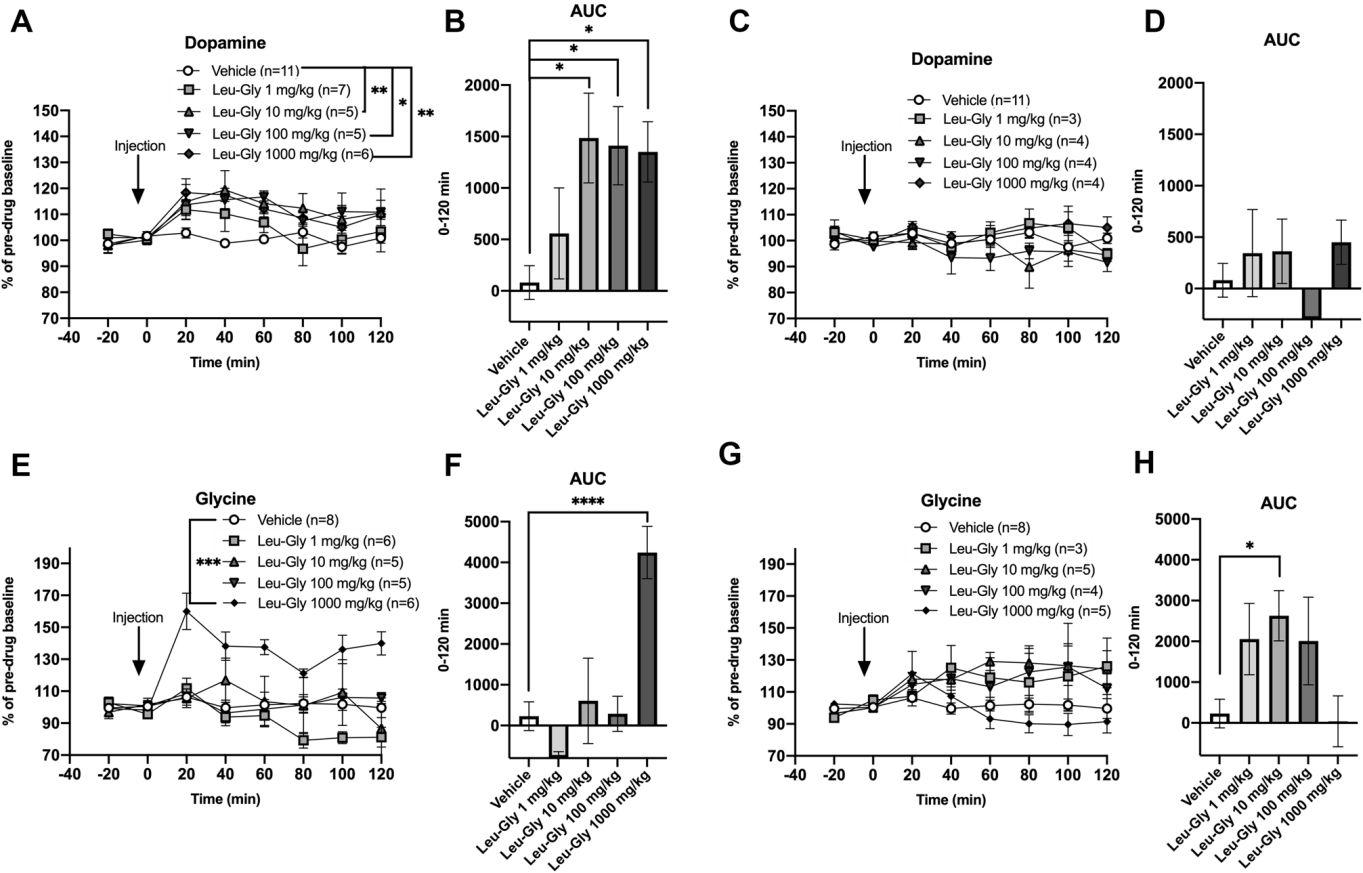
Systemic Leu-Gly elevates accumbal dopamine levels in a subpopulation of rats. **A** In Leu-Gly-treated rats defined as dopamine responders, systemic administration of Leu-Gly (i.p.) significantly increased accumbal dopamine levels, as compared to vehicle-treated controls. In the lower dose-range (1–10 mg/kg) but not in the higher dose-range studied (10–1000 mg/kg) the effect appeared to be dose-related. **B** AUCs differed significantly between rats treated with Leu-Gly i.p. and vehicle-treated controls. (**C**–**D**) Upon comparison of accumbal dopamine levels between Leu-Gly-treated rats defined as dopamine non-responders and vehicle-treated controls, no significant treatment effects could be revealed. (**E**–**F**) In dopamine responding rats, a significant increase in nAc glycine levels was observed following treatment with the highest dose of Leu-Gly (1000 mg/kg). (**G**–**H**) However, also for dopamine non-responding rats treated with a lower dose of Leu-Gly (10 mg/kg), a slight increase in accumbal glycine levels was observed for comparisons of AUC but not for two-way ANOVA. Shown are mean values ± SEM, number of rats, *p < .05, **p < .01, ***p < 0.001, ****p < 0.0001

**Fig. 9:**
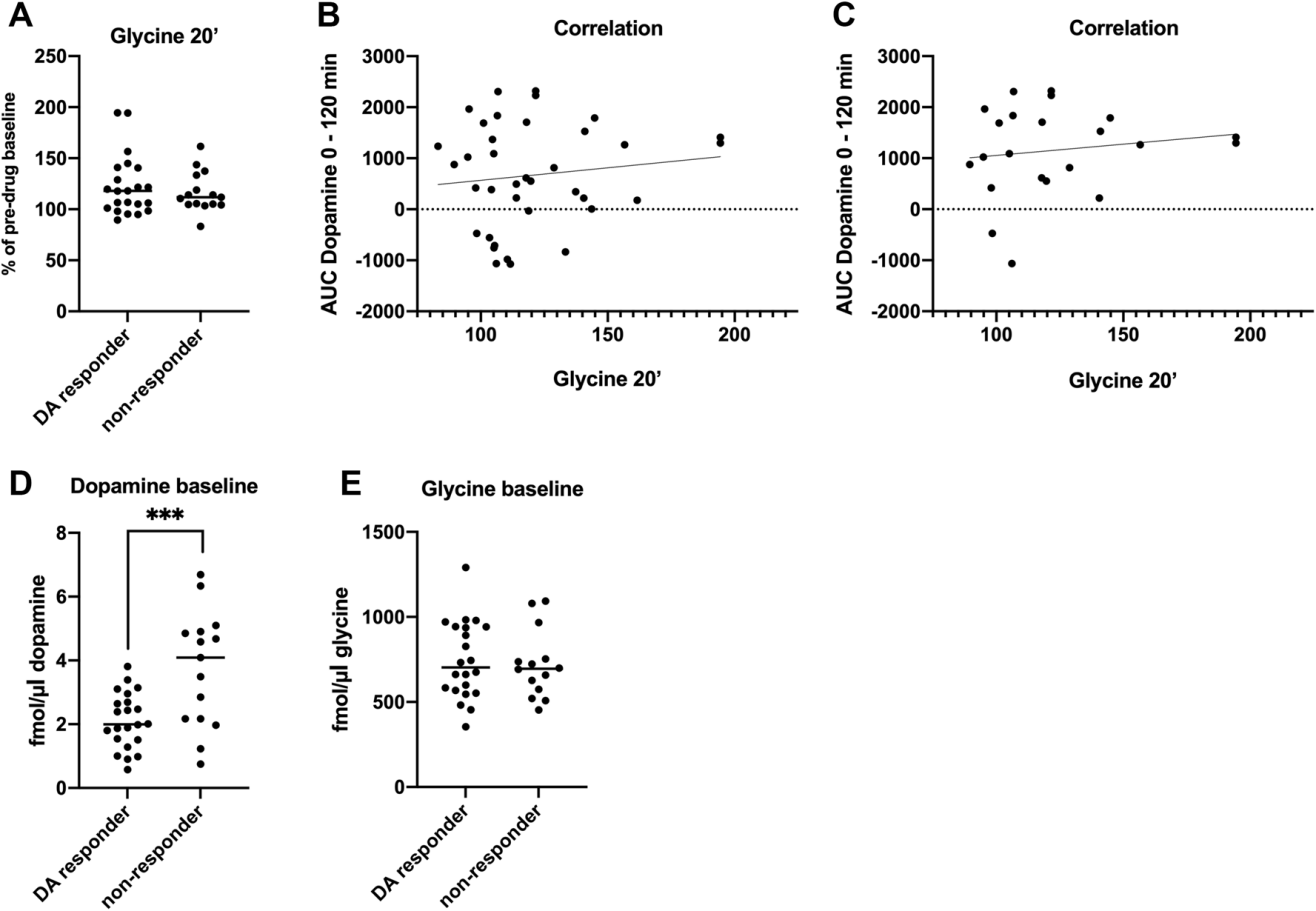
Lower baseline dopamine levels among dopamine responding rats. **A** Glycine peak levels at timepoint 20 min post injection did not differ between rats classified as dopamine responders vs non-responders. Comparisons of glycine peak levels (20’) vs a composite measure for dopamine output (AUC dopamine 0–120 min) did not reveal any significant relationships for all rats (**B**) or dopamine responders alone (**C**). (**D**–**E**) Rats classified as dopamine responders presented significantly lower basal dopamine levels compared to non-responding rats, whereas no differences were observed for basal glycine levels. Shown are individual and mean values, ***p < .001

The accumbal dopamine response for each individual rat was scrutinized to investigate whether a subpopulation of animals presented elevated extracellular dopamine levels of more than 10%, a heterogenous response that has previously been observed following glycine administered i.p. or locally into the nAc (Molander and Soderpalm, 2005a, Olsson et al. 2020). As depicted in Fig. 7, a subset of rats was identified as dopamine responders and the proportion of responding versus non-responding rats was similar across doses of Leu-Gly; 1 mg/kg (7/10), 10 mg/kg (5/9), 100 mg/kg (5/9) and 1000 mg/kg (6/10). On the basis of this finding, results are also presented for dopamine responders alone. A two-way-ANOVA revealed a significant elevation of accumbal dopamine; treatment effect *F*_(429)_ = 5.38, *p* = 0.002, time effect *F*_(6174)_ = 6.46, *p* < 0.001, interaction term *F*_(24,174)_ = 1.07, *p* = 0.380. Dunnett’s post hoc revealed significant effects produced by the three highest doses, Leu-Gly 10 mg/kg *vs* vehicle *p* = 0.007, Leu-Gly 100 mg/kg *vs* vehicle *p* = 0.010 and Leu-Gly 1000 mg/kg *vs* vehicle *p* = 0.009 (Fig. 8A). Likewise, the AUC was significantly increased in rats treated with the higher doses; one-way-ANOVA *F*_(429)_ = 4.31, *p* = 0.007, Dunnett’s post hoc Leu-Gly 10 mg/kg *vs* vehicle *p* = 0.016, Leu-Gly 100 mg/kg *vs* vehicle *p* = 0.024, Leu-Gly 1000 mg/kg vs vehicle *p* = 0.021 (Fig. 8B). In contrast, dopamine levels were not altered among the dopamine non-responding rats (Fig. 8C–D). In an attempt to characterize what distinguishes dopamine responders from non-responders, nAc glycine levels following treatment were also re-examined, based on dopamine response status. In dopamine responding rats, a distinct and prolonged elevation in nAc glycine output, reaching peak levels of approx. 55% at timepoint 20’ was observed following treatment with the highest dose, two-way ANOVA; treatment effect *F*_(425)_ = 4.77, *p* < 0.001, time effect *F*_(6150)_ = 6.70, *p* < 0.001, interaction term *F*_(24,150)_ = 3.33, *p* < 0.001, Dunnett’s post hoc Leu-Gly 1000 mg/kg *vs* vehicle *p* = 0.001 (Fig. 8E). These effects were also evident when comparing AUCs, one-way ANOVA *F*_(424)_ = 11.45, *p* < 0.0001, Dunnett’s post hoc Leu-Gly 1000 mg/kg vs vehicle* p* < 0.0001 (Fig. 8F). However, also dopamine non-responding rats presented a slight elevation in accumbal glycine following treatment with a lower dose of Leu-Gly (10 mg/kg) upon comparisons of AUCs, one-way ANOVA; *F*_(420)_ = 3.59, *p* = 0.023, Leu-Gly 10 mg/kg *vs* vehicle *p* = 0.029 (Fig. 8G–H). Moreover, glycine peak values at timepoint 20’ was compared for responders vs non-responders, but revealed no significant differences, as depicted in Fig. 9A. Further comparisons for peak glycine (20’) vs AUC dopamine for all rats and dopamine responding rats alone revealed no significant relationships (Fig. 9B–C). Also, baseline dopamine levels were compared for responders (2.11 ± 0.19 nM) vs non-responders (3.72 ± 0.47 nM), revealing a significantly lower accumbal dopamine tone in rats classified as responders, *t *(35) = 3.63, *p* < 0.001 (Fig. 9D). Baseline glycine levels did not differ between responding and non-responding rats (Fig. 9E).

## Discussion

Repeated findings from animal models suggest that compounds that elevate brain glycine levels may be implicated for the treatment of AUD and schizophrenia, targeting preferentially GlyRs in the nAc or the co-agonist site on NMDA-Rs in the prefrontal cortex and hippocampus, respectively (Balu 2016, Soderpalm et al. 2017). However, despite a robust preclinical rationale, the only glycinergic compound, a GlyT1-inhibitor, that has to-date been evaluated in a randomized controlled trial (RCT) for the treatment of AUD failed to demonstrate superiority to placebo (de Bejczy et al. 2014). Contrariwise, several RCTs with either GlyT1-inhibitors or high-dose glycine as add-on treatments to conventional antipsychotics in the treatment for schizophrenia have yielded beneficial outcomes but with variable clinical efficacy (Kaufman et al. 2009). The divergent clinical response to high-dose glycine therapy may be attributed to its inconsistent bioavailability and its impeded BBB passage, as demonstrated in clinical trials where participants present highly variable and at times presumably insufficient serum and brain glycine levels despite dosing adjusted for body weight (Heresco-Levy et al. 1999; D'Souza et al. 2000; Buchanan et al. 2007; Kaufman et al. 2009). Nevertheless, despite variable responses, glycine therapy at high doses indeed elevates CSF and brain glycine levels in humans, the latter measured non-invasively by MR-S. However, the individual CNS glycine response as measured by MR-S does not correlate to serum glycine increments, which underscores the need for a glycinergic compound that more readily passes the BBB and ultimately ensures a consistent delivery to the extracellular space (D'Souza et al. 2000; Kaufman et al. 2009).

On these premises, treatment with glycine anchored onto an amino acid that more readily passes the BBB, such as Leu-Gly that has previously been demonstrated to elevate whole brain tissue dopamine levels ex vivo, conceivably as a down-stream effect of nAc GlyR signaling, presented as a viable next step. In further support of this concept, it has previously been demonstrated that glycine-containing dipeptides pass the BBB (Tanaka et al. 2019). Similar to previously reported effects of Leu-Gly isomers (1, 10 mg/kg) on dopamine whole brain tissue levels, the present study revealed significantly raised accumbal dopamine tissue levels following Gly-Leu treatment (10 mg/kg). However, the remaining doses did not significantly alter accumbal glycine or dopamine levels as measured by either in vivo microdialysis or ex vivo neurochemistry. In the microdialysis study, trends for elevated glycine levels (approx. 30–50%) were observed following Leu-Gly 10 and 1000 mg/kg. Previous microdialysis studies with compounds that elevate central glycine levels suggest that peak increments of at least approx. 85% are required to yield an accumbal dopamine response or decrease ethanol intake and may explain why we did not observe any effects on dopamine levels measured in vivo (Molander et al. 2007; Lidö et al. 2009).

Notably, when previously administering a GlyT1-inhibitor i.p. or glycine itself either i.p. or locally into the nAc, only a subpopulation of the rats displayed a distinct dopamine response, i.e. a dopamine elevation by 10% or more compared to baseline during the first hour after injection, hence motivating a division between dopamine responders and non-responders (Molander and Soderpalm 2005a; Lidö et al. 2009; Olsson et al. 2020). Further, rats with dopamine elevation in response to bilateral perfusion of glycine in the nAc reduced their ethanol intake, and rats with dopamine elevation in response to systemic treatment with a GlyT1-inhibitor presented an attenuated EtOH-induced dopamine response, effects that were not evident in dopamine non-responders (Molander et al. 2005; Lidö et al. 2009). Indeed, although the present study could not reveal any effects on accumbal dopamine levels when regarding all rats as a homogenous group, a small but significant dopamine elevation was discerned following treatment with all but the lowest dose of Leu-Gly i.p. in the subpopulation of rats classified as dopamine responders, as compared to vehicle-treated controls. This effect was observed even following 10 mg/kg, a considerably lower dose than that of glycine itself (400–800 mg/kg) producing a similar dopamine elevation (Olsson et al. 2020). Moreover, glycine levels in dopamine responding rats were significantly raised following Leu-Gly 1000 mg/kg, suggestive of a relationship between exogenously applied glycine, by means of Leu-Gly i.p., and a dopamine response, presumably mediated by activated GlyRs, although causation cannot be proved from the present study. Arguing against this hypothesis, glycine levels were unaltered by lower doses of Leu-Gly that produced a dopamine-response, whereas slightly elevated glycine levels were observed also following a lower dose of Leu-Gly in non-responding rats. Further, glycine peak levels did not differ between responders and non-responders and were not correlated to dopamine output. Moreover, since dipeptides have been demonstrated not only to intactly pass the BBB but also to accumulate in the mouse brain parenchyma in ex vivo models, another possibility could be that Leu-Gly itself, in its undegraded form, acts as an agonist or a positive allosteric modulator at the GlyR (Tanaka et al. 2019). High-resolution molecular imaging techniques using protein crystallography and recently cryoelectron microscopy have been able to position the glycine-molecule in relationship to residues in the ligand-binding pocket of the GlyR with a high, although not unambiguous certainty (Huang et al. 2015; Kumar et al. 2020). It has been suggested that several parts of the glycine molecule engage in a hydrogen-bonding network with a number of residues in the GlyR, where the glycine carboxylate-group is of critical importance for binding to the ligand-pocket (Kasaragod and Schindelin 2018). The glycine carboxylate-group is indeed conserved in the Leu-Gly molecule, which lends some theoretical support for an interaction with the ligand-pocket. In future studies, it would be of great interest to determine the extracellular levels of Leu-Gly itself and their relationship to dopamine output.

Interestingly, when comparing baseline dopamine levels of dopamine responding and non-responding rats, rats classified as responders exhibited a significantly lower basal dopamine tone. It could be argued that larger percental fluctuations for rats presenting a low basal dopamine tone may merely reflect a methodological artifact. On the other hand, a low, endogenous dopamine tone in the nAc has previously been associated with high ethanol-intake in rats and may be an important determinant of alcohol drinking behavior (Weiss et al. 1993; Ericson et al. 2020). Thus, another interpretation of current and previous findings would be that a low, endogenous dopamine tone in the nAc represents a distinct endophenotype characterized by high ethanol intake and a dopamine response to glycine treatment (Lidö 2011). Since the GlyR antagonist strychnine concentration-dependently reduces dopamine output in the nAc, GlyR activation probably sustains basal dopamine levels in the rat nAc (Molander and Soderpalm 2005). It follows that reduced GlyR activation resulting from e.g*.* low ongoing stimulation of ligands at these receptors or neighboring G-protein-coupled receptors with intracellular signaling pathways modulating GlyR function (Yevenes et al. 2003) may result in low baseline dopamine levels. Moreover, differences in ongoing activity at the receptor, sub-unit constellations of the GlyR and/or desensitization phenomena may also influence the responsivity of the receptor, in analogy with observations made in other cys-loop ion-channel receptors (Fenster et al. 1997; Raltschev et al. 2016). Nonetheless, ongoing stimulation of ligands, as appraised by basal levels of extracellular glycine, did not differ between dopamine responders and non-responders in the present study. On the other hand, basal glycine levels measured by microdialysis may reflect the large metabolic pool of glycine rather than the neurotransmitter pool and other endogenous ligands or allosteric modulators of the GlyR not measured in the current study probably also influence the equilibrium between the activated, desensitized and resting states of the receptor (Kumar et al. 2020).

The smaller divergencies compared to the findings of Han et al. 2018 may be explained by differences in the study design, where Han et al. could identify elevated dopamine levels ex vivo in mice after subchronic, oral treatment whereas our study entailed acute, i.p. treatment with Leu-Gly isomers. Moreover, it is also conceivable that the previously observed whole brain dopamine elevation in mice was driven by changes in brain areas that were not investigated in the current study, i.e. outside the striatum or brainstem. It should further be noted that dipeptides are readily distributed from the gut mucosa to the blood circulation but are also subjected to hydrolysis into free amino acids in the intestinal lumen and in the blood stream (Rohm et al. 2019). Thus, it could be argued that both the present and previous studies of Leu-Gly isomers to a varying extent may also reflect the effects of systemic treatment with glycine and leucine themselves. It would therefore be advised to obtain also serum samples in further studies on CNS effects of systemically administered dipeptides. Notably, the highest dose of Leu-Gly, 1000 mg/kg, would if degraded into its constituent amino acids reflect a dose of systemic glycine that has previously shown a trend for elevated nAc glycine levels, in similarity to what was observed in the present study (Olsson et al. 2020). Moreover, the free amino acid glycine, including Leu-Gly following hydrolysis, is beside acting as a neurotransmitter also utilized in peripheral and CNS metabolism (Verleysdonk et al. 1999; Wang et al. 2013). Hence, despite a presumed, facilitated BBB passage for Leu-Gly, its availability for target GlyRs in the nAc may be compromised.

The underlying rational behind the locomotor activity study was to explore whether Leu-Gly interfered with animal behavior associated with ethanol-induced activation of central dopaminergic systems. In line with the psychomotor stimulant theory of addiction, most substances that interact with the mesolimbic dopamine system and are addictive, e.g. ethanol and amphetamine, may increase locomotion (Wise and Bozarth 1987), as also observed here following ethanol. In the present study, treatment with neither Leu-Gly (1–1000 mg/kg) nor Gly-Leu (1–100 mg/kg) alone altered locomotor activity, arguing against stimulatory properties of these dipeptides. Furthermore, the ethanol-induced hyperlocomotion was not significantly altered by Leu-Gly. Altogether, the negative findings on locomotor behavior are congruent with the lack of a convincing dopamine response in the nAc, when regarding all animals as a homogenous group, as measured either in tissues ex vivo or in microdialysis in vivo (Figs. 4, 5, 6).

To conclude, this study shows that systemic treatment with Leu-Gly does not elevate accumbal dopamine and glycine levels in vivo when the animals are regarded as a homogenous group, nor interferes with a behavior associated with activation of the mesolimbic reward system. In a subset of rats denoted dopamine-responders, dopamine levels were significantly elevated by much lower doses of Leu-Gly than previously observed following glycine itself (Olsson et al. 2020). This effect did not correlate to accumbal glycine levels and could instead stem from GlyR modulation by Leu-Gly itself. Moreover, baseline dopamine levels were lower in the responding subpopulation of rats and may provide a hint of the mechanisms underlying the observed differences in dopamine response that probably involve low ongoing, basal GlyR activity. The tentative relationship between dopamine baseline and ensuing response to glycinergic treatment and presumptive direct interactions between glycine-containing dipeptides and the GlyR bear insights for refinement of the glycinergic treatment concept for AUD. In contrast to what was originally hypothesized, treatment with Leu-Gly and Gly-Leu instead of glycine itself did not appear to facilitate glycine BBB passage, although differently composed dipeptides such as e.g. Gly-Sarcosine, that has been shown to clearly traverse the BBB in mice tissue ex vivo, may in theory yield more favorable results (Tanaka et al. 2019). Nonetheless, the concept of treatment with a glycine-containing dipeptide, instead of glycine itself, to enhance accumbal GlyR signaling still appears interesting. Future attempts utilizing dipeptides would benefit from an improved understanding of its BBB passage and degradation. Finally, still at this point, GlyT1-inhibitors may be the preferable pharmacological agent for elevating central glycine levels as a way of interfering with the rewarding properties of ethanol and reduce ethanol intake. Targeting central glycine levels alone may, however, be insufficient to robustly interfere with ethanol’s reinforcing properties, since ethanol besides mesolimbic dopamine interferes with several different receptor systems in the reward circuit. Thus, future studies on glycinergic compounds as a pharmacotherapy for AUD would benefit from studying the effects of a combined treatment that targets several transmitter systems and signaling pathways including glycine and the GlyR.

## Data Availability

The datasets generated during and/or analyzed during the current study are available from the corresponding author on reasonable request.

## References

[CR1] Abbott NJ, Ronnback L, Hansson E (2006). Astrocyte-endothelial interactions at the blood-brain barrier. Nat Rev Neurosci.

[CR2] Balu DT (2016). The NMDA receptor and schizophrenia: from pathophysiology to treatment. Adv Pharmacol.

[CR3] Björklund A, Dunnett SB (2007). Dopamine neuron systems in the brain: an update. Trends Neurosci.

[CR4] Boileau I, Assaad JM, Pihl RO, Benkelfat C, Leyton M, Diksic M, Tremblay RE, Dagher A (2003). Alcohol promotes dopamine release in the human nucleus accumbens. Synapse.

[CR5] Buchanan RW, Javitt DC, Marder SR, Schooler NR, Gold JM, McMahon RP, Heresco-Levy U, Carpenter WT (2007). The cognitive and negative symptoms in schizophrenia trial (CONSIST): the efficacy of glutamatergic agents for negative symptoms and cognitive impairments. Am J Psychiatry.

[CR6] Carlsson A, Engel J, Strombom U, Svensson TH, Waldeck B (1974). Suppression by dopamine-agonists of the ethanol-induced stimulation of locomotor activity and brain dopamine synthesis. Naunyn Schmiedebergs Arch Pharmacol.

[CR7] de Bejczy, A., Nations, K. R., Szegedi, A., Schoemaker, J., Ruwe, F. & Soderpalm, B, 2014 de Bejczy, A., Nations, K. R., Szegedi, A., Schoemaker, J., Ruwe, F, Soderpalm, B. (2014). Efficacy and safety of the glycine transporter-1 inhibitor org 25935 for the prevention of relapse in alcohol-dependent patients: a randomized, double-blind, placebo-controlled trial. Alcohol Clin Exp Res 38:2427-2435 10.1111/acer.12501 25257291

[CR8] di Chiara G, Imperato A (1988). Drugs abused by humans preferentially increase synaptic dopamine concentrations in the mesolimbic system of freely moving rats. Proc Natl Acad Sci.

[CR9] D'Souza DC, Gil R, Cassello K, Morrissey K, Abi-Saab D, White J, Sturwold R, Bennett A, Karper LP, Zuzarte E, Charney DS, Krystal JH (2000). IV glycine and oral D-cycloserine effects on plasma and CSF amino acids in healthy humans. Biol Psychiatry.

[CR10] Engel JA, Fahlke C, Hulthe P, Hård E, Johannessen K, Snape B, Svensson L (1988). Biochemical and behavioral evidence for an interaction between ethanol and calcium channel antagonists. J Neural Transm.

[CR11] Ericson M, Ulenius L, Andrén A, Jonsson S, Adermark L, Söderpalm B (2020). Different dopamine tone in ethanol high- and low-consuming Wistar rats. Addict Biol.

[CR12] Fenster CP, Rains MF, Noerager B, Quick MW, Lester RA (1997). Influence of subunit composition on desensitization of neuronal acetylcholine receptors at low concentrations of nicotine. J Neurosci.

[CR13] Frye GD, Breese GR (1981). An evaluation of the locomotor stimulating action of ethanol in rats and mice. Psychopharmacology.

[CR14] Gonzales RA, Job MO, Doyon WM (2004). The role of mesolimbic dopamine in the development and maintenance of ethanol reinforcement. Pharmacol Ther.

[CR15] Han NR, Kim HY, Kim NR, Lee WK, Jeong H, Kim HM, Jeong HJ (2018). Leucine and glycine dipeptides of porcine placenta ameliorate physical fatigue through enhancing dopaminergic systems. Mol Med Rep.

[CR16] Hawkins RA, O'Kane RL, Simpson IA, Vina JR (2006). Structure of the blood-brain barrier and its role in the transport of amino acids. J Nutr.

[CR17] Heresco-Levy U, Javitt DC, Ermilov M, Mordel C, Silipo G, Lichtenstein M (1999). Efficacy of high-dose glycine in the treatment of enduring negative symptoms of schizophrenia. Arch Gen Psychiatry.

[CR18] Howard EC, Schier CJ, Wetzel JS, Gonzales RA (2009). The dopamine response in the nucleus accumbens core-shell border differs from that in the core and shell during operant ethanol self-administration. Alcohol Clin Exp Res.

[CR19] Huang X, Chen H, Michelsen K, Schneider S, Shaffer PL (2015). Crystal structure of human glycine receptor-α3 bound to antagonist strychnine. Nature.

[CR20] Kasaragod VB, Schindelin H (2018). Structure-function relationships of glycine and GABA(A) receptors and their interplay with the scaffolding protein gephyrin. Front Mol Neurosci.

[CR21] Kaufman MJ, Prescot AP, Ongur D, Evins AE, Barros TL, Medeiros CL, Covell J, Wang L, Fava M, Renshaw PF (2009). Oral glycine administration increases brain glycine/creatine ratios in men: a proton magnetic resonance spectroscopy study. Psychiatry Res.

[CR22] Koob GF, Volkow ND (2016). Neurobiology of addiction: a neurocircuitry analysis. The Lancet Psychiatry.

[CR23] Kumar A, Basak S, Rao S, Gicheru Y, Mayer ML, Sansom MSP, Chakrapani S (2020). Mechanisms of activation and desensitization of full-length glycine receptor in lipid nanodiscs. Nat Commun.

[CR24] Lidö HH, Stomberg R, Fagerberg A, Ericson M, Söderpalm B (2009). The glycine reuptake inhibitor org 25935 interacts with basal and ethanol-induced dopamine release in rat nucleus accumbens. Alcoholism: Clin Exp Res.

[CR25] Lidö HH, Marston H, Ericson M, Söderpalm B (2012). The glycine reuptake inhibitor Org24598 and acamprosate reduce ethanol intake in the rat; tolerance development to acamprosate but not to Org24598. Addict Biol.

[CR26] Lidö, H. H. 2011. Preclinical investigations of GlyT-1 inhibition as a new concept for treatment of alcohol dependence. Doctoral thesis, institute of neuroscience and physiology at sahlgrenska academy, University of Gothenburg, Sweden.

[CR27] Liljequist S, Berggren U, Engel J (1981). The effect of catecholamine receptor antagonists on ethanol-induced locomotor stimulation. J Neural Transm.

[CR28] LYNCH, J. W.  (2004). Molecular structure and function of the glycine receptor chloride channel. Physiol Rev.

[CR29] Molander A, Soderpalm B (2005). Glycine receptors regulate dopamine release in the rat nucleus accumbens. Alcohol Clin Exp Res.

[CR30] Molander A, Söderpalm B (2005). Accumbal strychnine-sensitive glycine receptors: an access point for ethanol to the brain reward system. Alcoholism: Clin Exp Res.

[CR31] Molander A, Lof E, Stomberg R, Ericson M, Soderpalm B (2005). Involvement of accumbal glycine receptors in the regulation of voluntary ethanol intake in the rat. Alcohol Clin Exp Res.

[CR32] Molander A, Lidö HH, Löf E, Ericson M, Söderpalm B (2007). The glycine reuptake inhibitor Org 25935 decreases ethanol intake and preference in male wistar rats. Alcohol Alcohol.

[CR33] Nestler EJ (2005). Is there a common molecular pathway for addiction?. Nat Neurosci.

[CR34] Olsson Y, Höifödt Lidö H, Danielsson K, Ericson M, Söderpalm B (2020). Effects of systemic glycine on accumbal glycine and dopamine levels and ethanol intake in male Wistar rats. J Neural Transm.

[CR35] Oxender DL, Christensen HN (1963). Distinct mediating systems for the transport of neutral amino acids by the Ehrlich cell. J Biol Chem.

[CR36] Raltschev C, Hetsch F, Winkelmann A, Meier JC, Semtner M (2016). Electrophysiological signature of homomeric and heteromeric glycine receptor channels. J Biol Chem.

[CR37] Rohm F, Skurk T, Daniel H, Spanier B (2019). Appearance of Di- and tripeptides in human plasma after a protein meal does not correlate with PEPT1 substrate selectivity. Mol Nutr Food Res.

[CR38] Rossetti ZL, Melis F, Carboni S, Diana M, Gessa GL (1992). Alcohol withdrawal in rats is associated with a marked fall in extraneuronal dopamine. Alcohol Clin Exp Res.

[CR39] Soderpalm B, Lido HH, Ericson M (2017). The glycine receptor-a functionally important primary brain target of ethanol. Alcohol Clin Exp Res.

[CR40] Spanagel R (2009). Alcoholism: a systems approach from molecular physiology to addictive behavior. Physiol Rev.

[CR41] Tanaka M, Dohgu S, Komabayashi G, Kiyohara H, Takata F, Kataoka Y, Nirasawa T, Maebuchi M, Matsui T (2019). Brain-transportable dipeptides across the blood-brain barrier in mice. Sci Rep.

[CR42] Toth E, Lajtha A (1981). Elevation of cerebral levels of nonessential amino acids in vivo by administration of large doses. Neurochem Res.

[CR43] Uchino H, Kanai Y, Kim DK, Wempe MF, Chairoungdua A, Morimoto E, Anders MW, Endou H (2002). Transport of amino acid-related compounds mediated by L-type amino acid transporter 1 (LAT1): insights into the mechanisms of substrate recognition. Mol Pharmacol.

[CR44] Ulenius L, Adermark L, Söderpalm B, Ericson M (2019). Energy drink constituents (caffeine and taurine) selectively potentiate ethanol-induced locomotion in mice. Pharmacol Biochem Behav.

[CR45] Ulenius L, Andrén A, Adermark L, Söderpalm B, Ericson M (2020). Sub-chronic taurine administration induces behavioral sensitization but does not influence ethanol-induced dopamine release in the nucleus accumbens. Pharmacol Biochem Behav.

[CR46] Vengeliene V, Leonardi-Essmann F, Sommer WH, Marston HM, Spanagel R (2010). Glycine transporter-1 blockade leads to persistently reduced relapse-like alcohol drinking in rats. Biol Psychiat.

[CR47] Vengeliene V, Rossmanith M, Takahashi TT, Alberati D, Behl B, Bespalov A, Spanagel R (2018). Targeting glycine reuptake in alcohol seeking and relapse. J Pharmacol Exp Ther.

[CR48] Verleysdonk S, Martin H, Willker W, Leibfritz D, Hamprecht B (1999). Rapid uptake and degradation of glycine by astroglial cells in culture: synthesis and release of serine and lactate. Glia.

[CR49] Volkow ND, Wang GJ, Telang F, Fowler JS, Logan J, Jayne M, Ma Y, Pradhan K, Wong C (2007). Profound decreases in dopamine release in striatum in detoxified alcoholics: possible orbitofrontal involvement. J Neurosci.

[CR50] Volkow ND, Michaelides M, Baler R (2019). The Neuroscience of drug reward and addiction. Physiol Rev.

[CR51] Wang W, Wu Z, Dai Z, Yang Y, Wang J, Wu G (2013). Glycine metabolism in animals and humans: implications for nutrition and health. Amino Acids.

[CR52] Weiss F, Lorang MT, Bloom FE, Koob GF (1993). Oral alcohol self-administration stimulates dopamine release in the rat nucleus accumbens: genetic and motivational determinants. J Pharmacol Exp Ther.

[CR53] Wise RA, Bozarth MA (1987). A psychomotor stimulant theory of addiction. Psychol Rev.

[CR54] Yevenes GE, Peoples RW, Tapia JC, Parodi J, Soto X, Olate J, Aguayo LG (2003). Modulation of glycine-activated ion channel function by G-protein betagamma subunits. Nat Neurosci.

[CR55] Yoder KK, Constantinescu CC, Kareken DA, Normandin MD, Cheng TE, O'Connor SJ, Morris ED (2007). Heterogeneous effects of alcohol on dopamine release in the striatum: a PET study. Alcohol Clin Exp Res.

